# Applicability of Small-Molecule Inhibitors in the Study of Peptidyl Arginine Deiminase 2 (PAD2) and PAD4

**DOI:** 10.3389/fimmu.2021.716250

**Published:** 2021-10-19

**Authors:** María Teresa Martín Monreal, Alexandra Stripp Rebak, Laura Massarenti, Santanu Mondal, Ladislav Šenolt, Niels Ødum, Michael L. Nielsen, Paul R. Thompson, Claus H. Nielsen, Dres Damgaard

**Affiliations:** ^1^ Institute for Inflammation Research, Center for Rheumatology and Spine Diseases, Rigshospitalet, Copenhagen University Hospital, Copenhagen, Denmark; ^2^ Department of Proteomics, Novo Nordisk Foundation Center for Protein Research (NNF CPR), Faculty of Health and Medical Sciences, University of Copenhagen, Copenhagen, Denmark; ^3^ Department of Biochemistry and Pharmacology, University of Massachusetts Medical School, Worcester, MA, United States; ^4^ Institute of Rheumatology and Department of Rheumatology, 1st Faculty of Medicine, Charles University, Prague, Czechia; ^5^ LEO Foundation Skin Immunology Research Center, Department of Immunology and Microbiology, University of Copenhagen, Copenhagen, Denmark; ^6^ Section for Periodontology, Department of Odontology, Faculty of Health and Medical Sciences, University of Copenhagen, Copenhagen, Denmark

**Keywords:** peptidyl arginine deiminase (PAD), citrullination, small-molecule PAD inhibitors, cell viability, rheumatoid arthritis

## Abstract

Citrullination, the conversion of peptidyl-arginine into peptidyl-citrulline, is involved in the breakage of self-tolerance in anti-CCP-positive rheumatoid arthritis. This reaction is catalyzed by peptidyl arginine deiminases (PADs), of which PAD2 and PAD4 are thought to play key pathogenic roles. Small-molecule PAD inhibitors such as the pan-PAD inhibitor BB-Cl-amidine, the PAD2-specific inhibitor AFM-30a, and the PAD4-specific inhibitor GSK199 hold therapeutic potential and are useful tools in studies of citrullination. Using an ELISA based on the citrullination of fibrinogen, we found that AFM-30a inhibited the catalytic activity of PADs derived from live PMNs or lysed PBMCs and PMNs and of PADs in cell-free synovial fluid samples from RA patients, while GSK199 had minor effects. In combination, AFM-30a and GSK199 inhibited total intracellular citrullination and citrullination of histone H3 in PBMCs, as determined by Western blotting. They were essentially nontoxic to CD4^+^ T cells, CD8^+^ T cells, B cells, NK cells, and monocytes at concentrations ranging from 1 to 20 μM, while BB-Cl-amidine was cytotoxic at concentrations above 1 μM, as assessed by flow cytometric viability staining and by measurement of lactate dehydrogenase released from dying cells. In conclusion, AFM-30a is an efficient inhibitor of PAD2 derived from PBMCs, PMNs, or synovial fluid. AFM-30a and GSK199 can be used in combination for inhibition of PAD activity associated with PBMCs but without the cytotoxic effect of BB-Cl-amidine. This suggests that AFM-30a and GSK199 may have fewer off-target effects than BB-Cl-amidine and therefore hold greater therapeutic potential.

## Introduction

Citrullination, also known as deimination, is the posttranslational conversion of peptidyl-arginine into peptidyl-citrulline in a calcium-dependent manner. Citrullination is catalyzed by the enzyme peptidyl arginine deiminase (PAD), of which five isozymes, PAD1-4 and -6, exist in humans (1). All PAD enzyme genes are encoded in chromosome 1, and PADs are usually located in the cytoplasm of the cell, except for PAD4, which has a nuclear localization sequence (2). Yet, PAD2 can also translocate into the nucleus upon calcium activation (3). Citrullination does not only play important physiological roles, e.g., in the central nervous system (4), in the skin (5), and in gene regulation (6) but also plays a pathogenic role by inducing autoantibody production in rheumatoid arthritis (RA) (7, 8). In addition, increased citrullination is associated with several other inflammatory diseases, including certain neurodegenerative diseases (9, 10) and different types of cancer (11–13).

PAD2 and PAD4 are expressed by various cell types of haematopoietic origin, including peripheral blood mononuclear cells (PBMCs) (14, 15), neutrophils (16), fibroblast-like cells (17) and osteoclasts (18). They are the most widely studied isoforms because of their expression in immune cells and inflamed tissues (19) such as the periodontium of subjects with periodontitis (17, 20), the synovium of RA patients (15, 21), and the alveolar tissue of smokers (22, 23). It has been a matter of some controversy, which of the two isoforms plays the most important role in catalyzing citrullination within and in the vicinity of polymorphonuclear leukocytes (PMNs) or PBMCs, as well as in the joints of RA patients (24–27).

Several small molecule PAD inhibitors derived from benzoyl-arginine compounds have been synthesized. The first- and second-generation pan-PAD inhibitors, Cl-amidine and BB-Cl-amidine respectively, are frequently used to study PAD function, both *ex vivo* and *in vivo* (28–30). PAD inhibitors have demonstrated remarkable efficacy in animal models of human disease including collagen-induced arthritis (CIA) (31), murine models of colitis (32), and lupus (33). Moreover, PAD inhibitors block the formation of neutrophil extracellular traps (NETs) (34). BB-Cl-amidine retains the critical elements of Cl-amidine but has some structural modifications that increase its plasma half-life and facilitate its cellular uptake. Even though BB-Cl-amidine inhibits citrullination by both PAD2 and PAD4, it has a preference for the latter (28).

Isoform-specific PAD inhibitors have also been developed (28). AFM-30a is a PAD2-specific inhibitor (35) while GSK199 is a PAD4-specific inhibitor which has shown modest efficacy in the CIA model of RA (36). Both inhibitors have shown an effect on IFN-γ production at both the mRNA and protein level in TLR-7-dependent lupus in mice (37). Yet, studies addressing the specificity and toxicity of these inhibitors in cellular systems are limited.

In the present study, we sought to address these issues. To that end, we evaluated both, intracellular and extracellular, PAD2 and PAD4 activities in PBMC and PMN cultures, as well as in cell-free synovial fluid from RA patients *via* PAD inhibition with AFM-30a, GSK199, and BB-Cl-amidine. Moreover, we examined the relative toxicities of the aforementioned PAD inhibitors in both PBMC and PMN cultures.

## Materials and Methods

### PBMCs From Healthy Donors and Cell-Free Synovial Fluid From RA Patients

This study included a total of six self-reported healthy, anonymous donors from the Center for Rheumatology and Spine Diseases at Copenhagen University Hospital, Rigshospitalet, Denmark. The study also included seven RA patients fulfilling the American College of Rheumatology criteria (38) from whom synovial fluid samples were obtained during knee joint aspirations. All seven patients were anti-CCP positive and presented antibodies against citrullinated fibrinogen. Synovial fluid samples were pretreated with hyaluronidase (Hylase^®^ Dessau; Riemser, Greifswald, Germany) for 30 min at 37°C and spun at 1,500×*g* for 10 min. Hyaluronidase-treated synovial fluid samples were then spun at 1,900×*g* for 10 min to ensure removal of leftover cells and debris and stored at −80°C until use. Collection of such biomaterial was approved by the local ethics committee of the Institute of Rheumatology in Prague, Czechia (No. 3294/2012). Besides, written informed consent was obtained from all patients.

### Isolation of Peripheral Blood Mononuclear Cells and Polymorphonuclear Leukocytes

Blood from six healthy donors was collected in heparin tubes (cat. 368480; BD Biosciences, Franklin Lakes, NJ, USA). For PBMC separation, blood was diluted 1:4 in phosphate-buffered saline (PBS) and transferred to tubes containing Lymphoprep™ (cat. 111457; Alere Technologies). The mixture was spun for 20 min, at 1,200×*g*, without brake, and the phase containing PBMCs was washed twice in PBS supplemented with 2% fetal bovine serum (FBS) (v/v) (cat. F9665, Sigma-Aldrich, St. Louis, MO, USA). PBMCs were then counted in a NucleoCounter^®^ NC-100™ device (cat. 900-0004; Chemometec, Allerød, Denmark). After, PBMCs were either lysed or resuspended to a concentration of 5 × 10^6^ cells/ml in RPMI 1640 (cat. 01-106-1a; Lonza, Basel, Switzerland) supplemented with 30% FBS and 10% DMSO (v/v) for cryopreservation. Cells were frozen at −80°C overnight and transferred to liquid nitrogen tanks the next day. Before use, 5 × 10^6^ PBMC aliquots were thawed by resuspending in RPMI supplemented with 10% (v/v) normal human serum (NHS), which is pooled serum from blood group AB-positive donors, (cat. H4522; Sigma-Aldrich).

For PMN separation, blood was transferred to 13 ml tubes and incubated with Dextran T500 5% in 0.9% NaCl in a proportion of 5:1 blood/dextran. After incubation for 1 h, the top leukocyte-enriched layer was transferred to a clean sterile tube containing Lymphoprep™, in 1:1 proportion, and spun at 1,200×*g* for 15 min without brake. The supernatant was discarded, and the pellet containing PMNs and red blood cells was resuspended in 1 ml of 0.2% NaCl solution. After 1 min, allowing for red blood cell lysis, 1 ml of 1.6% NaCl solution was added and the cells were spun again at 1,200×*g* for 5 min. The isolated PMNs were resuspended in PBS supplemented with 2% FBS (v/v) and counted, spun again, and either lysed or resuspended in RPMI supplemented with 10% (v/v) NHS and 0.1% (v/v) gentamycin.

### Preparation of PBMCs and PMNs lysates

2x10^6^ freshly isolated PBMCs or PMNs were lysed in 20 μl cell lysis buffer (cat. 9803, Cell Signaling Technology, Danvers, MA, USA) following manufacturer’s instructions. In order to measure PAD activity with the same amounts of PADs for both PBMCs and PMNs, total protein concentration was determined in each sample with a Pierce BCA protein assay kit (cat. 23225, Thermo Fisher, Waltham, MA, USA) according to the manufacturer’s instructions. Lysates were stored at −80°C until use.

### PAD Inhibitors

BB-Cl-amidine, AFM-30a, and GSK199 were synthesized as reported earlier in (33–35).

### Assay to Determine PAD Activity Using Fibrinogen as Substrate

PAD activity was determined using an ELISA-based assay with fibrinogen as substrate, as previously described in (39). Nunc Maxisorp plates were coated with 100 μl/well of 1 μg/ml fibrinogen (cat. 341578, Sigma Aldrich) and left overnight at 4°C. Wells were washed three times in washing buffer (PBS, 0.05% Tween-20, pH 7.4) and blocked in Tris-buffered saline (TBS) buffer containing 0.05% Tween-20, pH 7.4, for 20 min at room temperature (RT). Hereafter, wells were incubated with 30 ng/ml of recombinant human PAD2 (cat. 10785, Cayman Chemicals, Ann Arbor, MI, USA) or 150 ng/ml recombinant human PAD4 (cat. 10500, Cayman Chemicals) in citrullination buffer (100 mM Tris-HCl, 5 mM CaCl_2_, 1 mM DTT, pH 7.5) in the presence of increasing concentrations of the different PAD inhibitors (0.13–62.5 μM). DMSO was used as a vehicle control. For cellular experiments, cell lysates (~100 µg/ml for PMNs and ~30 µg/ml for PBMCs) or freshly isolated live cells (500,000 cells/well) were incubated in citrullination buffer with or without the inhibitors, and for synovial fluid experiments, synovial fluids were incubated 1:10 in citrullination buffer with or without the inhibitors. In lysates, cellular, and synovial fluid experiments, PAD inhibitors were used at the following concentrations: 20 µM BB-Cl-amidine, 20 µM AFM-30a, or 20 µM GSK199. EDTA was used at 25 mM concentration as a positive control of PAD inhibition. After three washes in washing buffer, wells were incubated for 90 min at RT with 0.5 μg/ml of murine anticitrullinated fibrinogen antibody (clone 20B2; cat. MQ13.102, ModiQuest, Oss, Netherlands). After three further washes, wells were incubated with 100 μl horse radish peroxidase-conjugated polyclonal rabbit-antimouse immunoglobulin antibody (cat. P0260; Dako, Glostrup, Denmark), diluted 1:1,000 in washing buffer. Finally, wells were washed three times in washing buffer and incubated with 1-Step Ultra TMB ELISA Substrate solution (cat. 34029, Thermo Fisher). After 10 min, the colorimetric reaction was stopped with 1 M H_2_SO_4_. Optical density (OD) was measured at 490–650 nm using the SPECTROstar nano Microplate Reader (BMG Labtech, Ortenberg, Germany), and data were processed using MARS software (BMG Labtech).

### Antimodified Citrulline and Histone H3 R2 Citrulline Western Blotting

PBMC and PMNs were isolated as described in ***Isolation of Peripheral Blood Mononuclear Cells and Polymorphonuclear Leukocytes*** and transferred to Lockes buffer (10 mM HEPES pH 7.5, 150 mM NaCl, 5 mM KCl, and 2 mM CaCl_2_, 0.1% glucose) at a concentration of 2 × 10^6^ cells/ml. PAD activity was inhibited by a 1-h preincubation at 37°C with 20 μM BB-Cl-amidine, 20 μM GSK199, 20 μM AFM-30a, or a combination of 20 μM GSK199 and 20 μM AFM-30a. The calcium ionophore A23187 (cat. C7522, Sigma Aldrich) was added to a concentration of 4 µM and incubated for 30 min at 37°C. Cells were then lysed in 2% SDS lysis buffer (50 mM Tris-HCl pH 8.5, 150 mM NaCl), and the lysate was further diluted to 1% SDS in a 2-mM MgCl_2_ solution to allow complete DNA shearing after the subsequent addition of benzonase (cat. E8263, Sigma Aldrich). Protein concentration was determined using the Pierce BCA protein assay kit (cat. 23225, Thermo Fisher) according to the manufacturer’s instructions, and SDS-PAGE was performed with equal amounts of total protein for each sample. Citrullination was detected with the antimodified citrulline (AMC) Detection Kit (cat. 17-347B, Merck) whereas histone H3 R2 citrullination was detected by standard Western blot analysis using an antihistone H3 (citrulline Arg2) antibody (cat. Ab176843, Abcam, Cambridge, UK). Western blot membranes were stained with the Pierce™ Reversible Protein Stain Kit for PDVF Membranes (cat. 24585, Thermo Fisher) following manufacturer’s instructions as a control of total protein loading as shown in **Supplementary Figure S1**. Chemiluminescence was captured in Hyperfilm™ ECL™ films (cat. GE28-9068-35, Sigma Aldrich) which were developed in a Kodak X-ray processor 2000. The relative fluorescence intensity of each condition was calculated using the software Image Studio (Li-Cor Biotechnology, Lincoln, NE, USA). Background noise was subtracted as the median value for the pixels of the background segment of each lane and fluorescence intensities were normalized to those obtained in the presence of ionophore but absence of inhibitors (=100%).

### Culturing of PBMCs and PMNs With Different PAD Inhibitors

Thawed PBMCs were washed twice in PBS supplemented with 2% FBS (v/v) and cultured in 96-well, round-bottomed plates; 250,000 PBMCs were cultured per well in a final volume of 200 μl RMPI 1640 supplemented with 10% (v/v) NHS and 0.1% (v/v) gentamycin. Besides, 250,000 PMNs were incubated per well in 96-well round-bottomed plates in a final volume of 200 μl of the same media.

BB-Cl-amidine, AFM-30a, GSK199, or the combination of AFM-30a and GSK199 were added to PBMC or PMN cultures at final concentrations in the well of 1, 5, 10, and 20 μM. Each well was supplemented with 2 μl of inhibitor dilution; therefore, 2 μl of DMSO was added to the cells as a vehicle control. PBMCs were incubated for 24, 48, and 72 h while PMNs were incubated with inhibitors for 4 and 24 h at 37°C, 5% CO_2_.

### Live and Dead Cell Staining for Flow Cytometry

After incubation with PAD inhibitors, the plates were spun for 5 min at 300×*g*, 4°C. Supernatants were collected and stored at −80°C for further analysis of lactate dehydrogenase (LDH) release, and cells were washed with 200 μl PBS supplemented with 2% FBS (v/v). Subsequently, cells were resuspended in a mix of 1 μl immunoglobulin for intravenous use (Ivig, cat. 034401, CSL Behring, King of Prussia, PA, USA) and 2 μl mouse serum (cat. M5905, Sigma-Aldrich) per well and stained with pretitrated Annexin V FITC (cat. 640906, BioLegend, San Diego, CA, USA), 7-AAD (cat. 420403, BioLegend), anti-CD14 PerCP (cat. 325632, BioLegend), anti-CD4 APC (cat. 300514; BioLegend), anti-CD16 Alexa Fluor 700 (cat. 560713, BD Biosciences), anti-CD8 Brilliant Violet 421 (cat. 562428, BD Biosciences), anti-CD3 Brilliant Violet 510 (cat. 564713, BD Biosciences), anti-CD56 PE (cat. 555516, BD Biosciences), and anti-CD19 PE-Cy7 (cat. 302216, BioLegend) for PBMCs and with pretitrated Annexin V FITC, 7-AAD, anti-CD15 PE-Cy7 (cat. 323030, BioLegend), and anti-CD45 APC (cat. 340910, BD Biosciences) for PMNs. After incubation for 20 min at 4°C, 100,000 cells per sample were acquired in an Attune™ NxT flow cytometer (Thermo Fisher Scientific). The gating strategy followed for identification of PBMC populations and PMNs is shown in **Supplementary Figures S2, S3**, respectively. Early apoptotic cells were defined as Annexin V FITC positive, 7-AAD negative while late apoptotic and necrotic cells were defined as Annexin V FITC positive, 7-AAD positive and Annexin V FITC-negative, 7-AAD-positive, respectively.

### Cytotoxicity Assessment by LDH Release

Cytotoxicity was assessed by quantification of LDH released from PBMCs and PMNs, using the CytoTox 96^®^ Non-Radioactive Cytotoxicity Assay (cat. G1780, Promega, Madison, WI, USA) according to the manufacturer’s instructions. In brief, 50 µl of culture supernatants were incubated with 50 µl of substrate solution containing tetrazolium salt (INT) and diaphorase for 30 min in the dark, and the coupled-enzymatic reaction was stopped by addition of 50 µl stop solution. Absorbance was read at 492 nm on a SPECTROstar nano Microplate Reader (BMG Labtech). Background OD values from wells containing only media were subtracted. Freshly isolated PBMCs or PMNs (250,000 cells/well), treated for 45 min with the cell lysis buffer of the kit, were used as positive controls for maximum LDH release. LDH presence in the supernatants was normalized to the LDH release in the positive controls.

### Statistics

Data are presented as the arithmetic mean ± standard deviation (SD). Differences between citrullination of human fibrinogen in presence and absence of the PAD inhibitors in cell lysates, live PMNs, and cell-free synovial fluid were evaluated using one-way analysis of variance (ANOVA) with Dunnett’s correction for multiple comparisons. The same applied for differences of the effect of the PAD inhibitors in total citrullination and histone H3 citrullination. *p*-Values <0.05 were considered statistically significant. Graphical representation of the data and statistics were performed with GraphPad Prism 9 software (GraphPad Software, San Diego, CA, USA).

## Results

### PAD-Inhibitor Specificity

**Figure 1:**
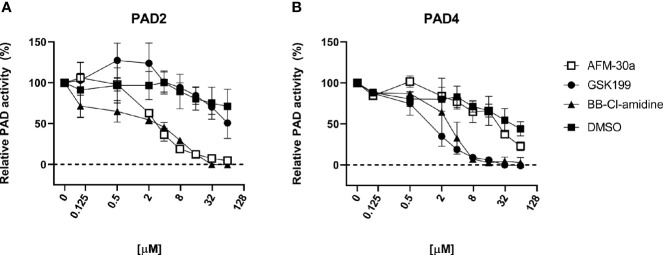
Inhibitory capacity of AFM-30a, GSK199, and BB-Cl-amidine on citrullination of fibrinogen. ELISA plates were coated with human fibrinogen. **(A)** Recombinant human PAD2 or **(B)** PAD4 were used for citrullination of fibrinogen in the presence of BB-Cl-amidine, AFM-30a, or GSK199 at various concentrations. DMSO was used as a vehicle control. Following a 2-h incubation, a monoclonal antibody (clone 20B2) recognizing a citrullinated epitope of fibrinogen was added to assess the extent of citrullination. PAD activity was normalized to that observed in the absence of inhibitors (=100%) and is expressed as the mean ± SD of duplicates.

Using human fibrinogen as substrate, we compared the capacity of BB-Cl-amidine, AFM-30a, and GSK199 to inhibit citrullination catalyzed by human recombinant PAD2 or PAD4 (**Figure 1**). As expected, AFM-30a inhibited PAD2-mediated citrullination in a concentration-dependent manner with 90% inhibition being observed at concentrations around 15 µM (**Figure 1A** **).** On the other hand, AFM-30a had negligible effect on PAD4-mediated citrullination unless used in concentrations well above 20 µM (**Figure 1B**).

Conversely, GSK199 inhibited PAD4-mediated citrullination in a dose-dependent manner with more than 90% inhibition being achieved at concentrations above 8 µM (**Figure 1B**), while it did not inhibit PAD2-mediated citrullination unless used in concentrations above 30 µM (**Figure 1A**). It should be noted that five times higher concentrations of PAD4 than of PAD2 were used to achieve comparable PAD activity in the assay employed.

As expected, BB-Cl-amidine inhibited both PAD isoforms in a dose-dependent manner with 90% inhibition of PAD2 and PAD4 observed at around 15–20 and 4 µM, respectively (**Figures 1A, B**).

### *Ex Vivo* Inhibition of PAD Activity

**Figure 2:**
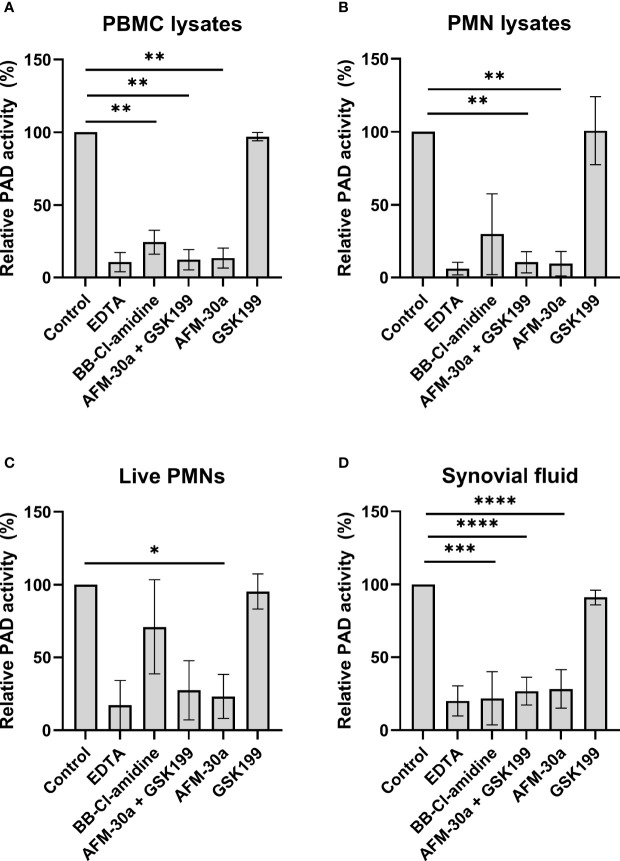
Effect of PAD inhibitors on leukocyte-derived PADs and on PADs in RA synovial fluid. ELISA plates were coated with human fibrinogen and incubated with **(A)** lysates from PBMCs from three healthy donors, **(B)** lysates from PMNs, **(C)** live PMNs isolated from the same three donors, or **(D)** synovial fluid from seven rheumatoid arthritis patients. Incubation took place in the presence of 25 mM EDTA, 20 µM BB-Cl-amidine, 20 µM AFM-30a, 20 µM GSK199, or a combination of the latter two. Untreated lysates, live cells or synovial fluid were used as controls. After 3 h of incubation, citrullination was detected with mAb 20B2 which recognizes a citrullinated epitope of fibrinogen. OD values were normalized to those observed in the absence of PAD inhibitor (=100%) and are shown as mean ± SD. ^*^ *p* < 0.05, ^**^ *p* < 0.01, ^***^ *P* < 0.001, and ^****^ *p* < 0.0001.

We next tested the inhibitory effects of these three compounds in human blood cell lysates and in cell-free synovial fluid. Cell lysates from PBMCs or PMNs were added to microtiter wells coated with fibrinogen, and PAD activity was assessed by ELISA (**Figure 2** and **Supplementary Figure S4**). Coincubation of PBMC (**Figure 2A**) or PMN lysates (**Figure 2B**) with the PAD2 inhibitor AFM-30a, alone or in combination with GSK199, inhibited citrullination to degrees comparable with those obtained with EDTA. By contrast, the PAD4-specific inhibitor, GSK199, showed limited effect compared with the nontreated control.

Next, we examined the effects of the inhibitors when live PMNs were added to fibrinogen-coated plates (**Figure 2C**). As described above for the cell lysates, incubation of live PMNs with AFM-30a significantly inhibited fibrinogen citrullination while the same tendency could be observed with the combination of the two specific PAD inhibitors. Incubation with BB-Cl-amidine or GSK199 showed modest effect, at best. A similar experiment was carried out for PBMCs, but the absorbance levels observed after 3 h of incubation were close to background levels (data not shown).

To test the activity of PAD2 and PAD4 contained in synovial fluid, cell-free synovial fluid samples from seven RA patients were incubated with fibrinogen in presence or absence of the inhibitors (**Figure 2D**). A pattern like that observed for the cell lysates, i.e., PAD2 being the essential catalyst, was observed.

### Effect of PAD Inhibitors on Intracellular Citrullination

To examine the ability of the PAD inhibitors to block intracellular PAD activity, we monitored changes in citrullination using both the AMC kit and a monoclonal antibody that recognizes histone H3 citrullinated at Arg2 (H3R2).

**Figure 3:**
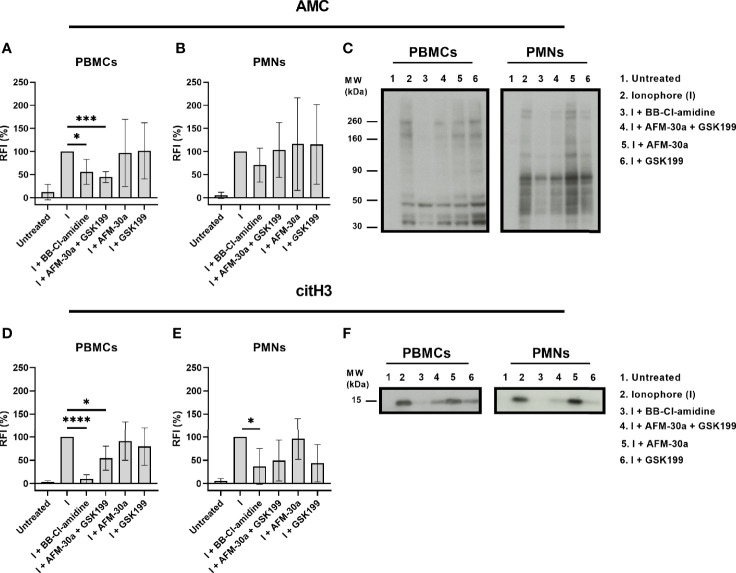
Inhibition of intracellular citrullination by small-molecule PAD inhibitors. Isolated PBMCs or PMNs from six healthy blood donors were preincubated with 20 μM of BB-Cl-amidine, 20 μM AFM-30a, 20 μM GSK199, or with a combination of the latter two for 1 h before addition of the calcium ionophore A23187. Untreated cells and ionophore-only treated cells (I) were used as negative and positive controls, respectively. The cells were then lysed, and the lysates were examined by Western blotting using the AMC method for visualization of total citrullination or a mAb for detection of histone H3 citrullination. Relative fluorescence intensities (RFI) were normalized to those obtained in the presence of ionophore but absence of inhibitors (=100%). **(A)** RFI (%) as a measurement of total protein citrullination in PBMC lysates and **(B)** PMN lysates. **(C)** Representative AMC blot for PBMC (left) and PMN lysates (right). **(D)** RFI (%) as a measurement of histone H3 citrullination in PBMC lysates and **(E)** PMN lysates. **(F)** Representative blot for citrullinated histone H3 in PBMC (left) and PMN lysates (right). RFI (%) are represented as mean ± SD. *p < 0.05, ***p < 0.001, and ****p < 0.0001.

In PBMCs, BB-Cl-amidine as well as the combination of AFM-30a and GSK199 inhibited citrullination of most of the proteins, albeit not totally (**Figures 3A**, left panel in **C**). The PAD2- and PAD4-specific inhibitors, AFM-30a and GSK199, did not affect the total citrullination levels significantly on their own (**Figures 3A**, left panel in **C**).

In PMNs, none of the inhibitors showed a significant inhibitory effect on protein citrullination. BB-Cl-amidine appeared to inhibit total citrullination (**Figures 3B**, right panel in **C**) while AFM-30a and GSK199 were not efficacious, neither when used separately nor in combination (**Figures 3B, C**, right panel).

Focusing on citrullination of histone H3 in PBMCs, both, BB-Cl-amidine and the combination of AFM-30a and GSK199, had significant inhibitory effects, which were not observed for the specific inhibitors alone (**Figures 3D**, left panel in **F**). In PMNs, only BB-Cl-amidine inhibited histone H3 citrullination significantly (**Figures 3E**, right panel in **F**).

### Influence of Small-Molecule PAD Inhibitors on Cell Viability

**Figure 4:**
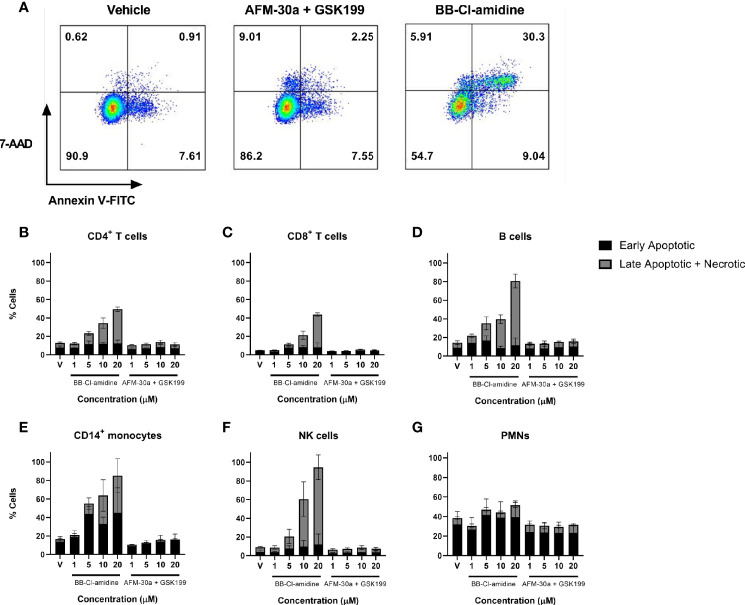
Effect of BB-Cl-amidine, AFM-30a, and GSK199 on cell survival. PBMCs and PMNs from three healthy donors were cultured in the presence of BB-Cl-amidine or a combination of AFM-30a and GSK199 at the given concentrations. DMSO was used as vehicle control (V). **(A)** After 24 h of incubation, the percentage of live and dead cells was assessed by flow cytometry after staining with Annexin V and 7-AAD as shown for CD4^+^ T cells in a representative sample. The percentages of early apoptotic and dead cells are shown for **(B)** CD4^+^ T cells, **(C)** CD8^+^ T cells, **(D)** B cells, **(E)** CD14^+^ monocytes, and **(F)** NK cells. **(G)** A similar staining was performed for PMNs incubated for 4 h with the inhibitors. Bars and error bars indicate mean ± SD.

When assessing the usefulness of PAD inhibitors in studies involving cell cultures, any effect on cell viability must be taken into consideration. We therefore quantified the percentage of live cells in PBMC or PMN cultures incubated with increasing concentrations of AFM-30a, GSK199, or BB-Cl-amidine. Viability of PBMCs was analyzed after 24, 48, and 72 h of incubation, and viability of PMNs was measured after 4 and 24 h. Among PBMCs, the populations analyzed were as follows: CD4^+^ T cells, CD8^+^ T cells, B cells, CD14^+^ monocytes, and NK cells. Early apoptotic cells were defined as Annexin V FITC positive, 7-AAD-negative whereas late apoptotic and necrotic cells were defined as Annexin V FITC positive and 7-AAD positive (**Figure 4A**). Annexin V FITC-negative and 7-AAD-positive cells were also included in this category.

While BB-Cl-amidine caused a reduction in viability of CD4^+^ T cells, CD8^+^ T cells, B cells, CD14^+^ monocytes, and NK cells within the 24-h observation period in a dose-dependent manner, the combination of AFM-30a and GSK199 had no effect on cell viability compared with the DMSO vehicle control (**Figures 4B–G**), neither did the two inhibitors used separately (data not shown). B cells (**Figure 4D**), CD14^+^ monocytes (**Figure 4E**) and NK cells (**Figure 3F**) were the populations showing the most pronounced cell death after incubation with BB-Cl-amidine. On the other hand, the level of apoptosis and cell death induced by BB-Cl-amidine and AFM-30a plus GSK199 was similar to the vehicle control in cultures of PMNs after 4 h of incubation (**Figure 4G**).

The percentages of live (Annexin V negative, 7-AAD negative) PBMCs at 48 and 72 h were largely unchanged from those observed after 24 h in the presence of different concentrations of BB-Cl-amidine, AFM-30a, and GSK199 in combination or the vehicle control. However, the proportion of cells in the late apoptotic and necrotic phases increased within this period (**Supplementary Figures S5A–E, S6**).

In the case of PMNs, the percentage of live cells at 24 h decreased compared with that observed at 4 h, whereas the percentage of dead cells increased under all conditions (**Supplementary Figure S5F**). Notably, there was a higher percentage of late apoptotic and necrotic cells in PMN cultures treated with 10 and 20 μM BB-Cl-amidine than in those treated with the same concentration of the combination of specific inhibitors.

**Figure 5:**
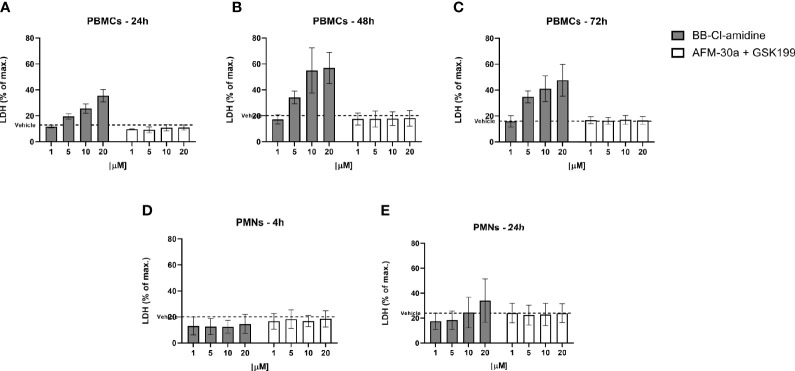
Effect of BB-Cl-amidine, AFM-30a, and GSK199 on LDH release. PBMCs or PMNs from three healthy donors were cultured in the presence of BB-Cl-amidine or a combination of AFM-30a and GSK199 at different concentrations. DMSO was used as vehicle control (broken line). LDH release was measured in PBMC culture supernatants after **(A)** 24 h, **(B)** 48 h, and **(C)** 72 h and in PMN culture supernatants after **(D)** 4 h and **(E)** 24 h. The LDH release was normalized to the LDH release occurring after total cell lysis (max. = 100%). Bars and error bars represent mean ± SD.

To further assess cell viability, we measured the LDH content leaked from dying cells into the culture supernatants. In accordance with the flow cytometric data, the release of LDH from PBMCs increased in a dose-dependent manner after treatment with BB-Cl-amidine, while the LDH release remained at background levels after treatment with the combination of AFM-30a and GSK199 (**Figure 5A**), or with the specific inhibitors alone (data not shown). A similar pattern of LDH release was observed in PBMC cultures incubated with PAD inhibitors for 48 and 72 h (**Figures 5B, C**).

Neither did BB-Cl-amidine, nor the combination of AFM-30a and GSK199, induce LDH release from PMNs (**Figure 5D**) during 4 h of incubation. Accordingly, the specific inhibitors did not affect LDH release on their own (data not shown). On the other hand, after 24 h of incubation with BB-Cl-amidine, there was an apparent trend towards induction of LDH release in a concentration-dependent manner, while the combination of specific inhibitors still had no effect (**Figure 5E**).

## Discussion

Small-molecule PAD inhibitors have shown promising effects as anti-inflammatory agents in experimental models of autoimmune diseases (28–36). However, off-target effects of the inhibitors are poorly elucidated, hereunder their toxic effects of individual types of immune cells. Moreover, it has been a matter of debate which PAD isoform, PAD2 or PAD4, is the most efficient catalyst mediating the citrullination within and in the vicinity of PMNs or PBMCs, and in synovial fluid of RA patients. In the present study, we address these issues.

To examine PAD activity, we used an assay detecting citrullination of a specific arginine residue in fibrinogen. When comparing PAD2 and PAD4 activities using this assay, it should be born in mind that the site in fibrinogen recognized by the developing antibody is citrullinated more efficiently by recombinant PAD2 than PAD4, hence we used five times higher concentrations of PAD4 than of PAD2 to achieve comparable OD values. Still, recombinant human PAD2 and recombinant human PAD4 both proved capable of citrullinating this site, and AFM-30a and GSK199 proved to be highly specific inhibitors of PAD2 and PAD4, respectively, which confirms previous findings (34, 35). Besides, both were as effective inhibitors of PADs as BB-Cl-amidine when used at concentrations below 30 μM.

Using the same fibrinogen-based assay, we examined the ability of AFM-30a and GSK199 to inhibit PADs present in PBMC or PMN lysates. While AFM-30a abrogated citrullination, GSK199 hardly had any effect. A similar pattern was observed when live PMNs were added to the fibrinogen-coated wells, suggesting that citrullination of the specific site in fibrinogen was performed by PAD2 associated with cell surfaces and/or secreted/leaked to the microenvironment. However, as stated above, PAD4 activity may have been underestimated in this respect due to the assay’s bias towards measurement of PAD2 activity.

Additionally, PAD2 contained in synovial fluid from RA patients also appeared to be mainly responsible for citrullination of the specific site in fibrinogen, in that PAD activity was abrogated by AFM-30a, while hardly being affected by GSK199. Although underestimation of PAD4-mediated citrullination may also apply in this case, a recent study showed similar results using antibody-mediated blockade of PAD2 secreted from neutrophils (40). Supporting a predominant role for PAD2 in the joints, a study on PAD2- and PAD4-deficient mice showed that PAD2, but not PAD4, is required for tumor necrosis factor-α-induced arthritis and citrullination in the inflamed joints (24).

Neither AFM-30a nor GSK199 *per se* appeared to inhibit citrullination of the bulk of proteins in PBMC lysates, as determined by Western blotting, but when used in combination, their effect was as great as that of BB-Cl-amidine, suggesting some intracellular proteins are predominantly citrullinated by PAD2, while others are citrullinated by PAD4. The latter are likely nuclear proteins, such as histone H3, since PAD4 is the intranuclear isoform (2).

It should be noted that due to the requirement of PADs for both calcium and reducing agents, we added exogenous calcium and DTT in the experiments concerning citrullination of fibrinogen, as well as exogenous calcium and a calcium ionophore in the experiments concerning total intracellular citrullination assessment and detection of citrullinated histone H3. Although universally employed in the literature, these conditions are not physiological and may have introduced some bias. In particular, the concentrations of the inhibitors needed to effectively inhibit citrullination may have been over- and underestimated. Indeed, GSK199 has higher affinity for the low-calcium state of PAD4 (34). Therefore, in the presence of a calcium ionophore and, consequently, exogenous calcium, PAD4 will presumably be in a high-calcium state, avoiding the binding of the PAD4-specific inhibitor. Hence, higher concentrations of the inhibitor might be needed to specifically inhibit PAD4 in this situation. Moreover, high calcium concentrations may favor the translocation of PAD2 to the nucleus (3), leading to an overestimation of any additional effect of PAD2 inhibition on histone H3 citrullination. Investigation of differential roles of PAD2 and PAD4 in cellular systems should preferably be performed without such additives.

It is imperative to ensure that any inhibitory effect of PAD inhibitors in functional cell studies is not due to off-target effects, especially cytotoxicity. We show in this study that BB-Cl-amidine is cytotoxic to T cells, B cells, monocytes, and NK cells when used in concentrations of 1 µM and above, while neither AFM-30a and GSK199 affected cell viability considerably at concentrations up to 20 µM where the inhibitory effect on PAD2 and PAD4 is strong. Cell viability was not even affected when the two compounds were used in combination and thereby amounted to a total concentration of 40 µM. Other studies have shown that BB-Cl-amidine is toxic to canine and feline cancer cell lines (29) and to T-helper cells derived from mice with collagen induced arthritis (30). However, it was claimed that the toxicity effects were specific for cancer cells or proinflammatory immune cells, respectively. The present study indicates that the toxicity also applies to PBMCs and PMNs from healthy donors even at low concentrations compared with those needed for inhibition of PADs.

In conclusion, this study confirms the specificity of the PAD inhibitors AFM-30a and GSK199 in different experimental set ups and shows that unlike the widely used pan-inhibitor BB-Cl-amidine, they are essentially nontoxic. Small-molecule PAD inhibitors are regarded as promising drug candidates for treatment of RA, and ongoing investigations aim at applying them to other diseases where citrullination may play a role, such as inflammatory bowel disease, multiple sclerosis, and systemic lupus erythematosus. Our data point toward targeting both isozymes in diseases associated with aberrant PAD activity, and our finding that specific PAD inhibitors are considerably less toxic than the pan-PAD inhibitor BB-Cl-amidine is important in that regard.

## Data Availability Statement

The raw data supporting the conclusions of this article will be made available by the authors, without undue reservation.

## Ethics Statement

The studies involving human participants were reviewed and approved by the Ethical Committee for the Capital Region of Denmark and the Institutional Ethics Committee of Institute of Rheumatology, Prague, Czechia (No. 3294/2012). The patients/participants provided their written informed consent to participate in this study.

## Author Contributions

MM, LM, CN, and DD designed the study. MM, AR, LM, and DD carried out the experiments. MM carried out the statistical analyses. MM, LM, DD, and CN drafted the manuscript. LŠ provided the synovial fluid samples. SM and PT provided the small-molecule PAD inhibitors. AR, SM, LŠ, NØ, MN, and PT revised the manuscript critically. All authors read and approved the final version.

## Funding

This work was supported by the University of Copenhagen and the Novo Nordisk Foundation Center for Protein Research as well as by the Danish Rheumatism Association (Grant number: A6760) together with the Ministry of Health of the Czech Republic (Research project number: 023728) and the Novo Nordisk Foundation (Grant number: NNF14CC0001 and NNF17CC0026748).

## Conflict of Interest

PRT holds several patents related to the development of PAD inhibitors and is a founder of Padlock Therapeutics, a wholly owned subsidiary of Bristol Myers Squibb from which he is entitled to milestone payments. PRT is a consultant for Related Sciences, a venture creation firm.

The remaining authors declare that the research was conducted in the absence of any commercial or financial relationships that could be construed as a potential conflict of interest.

## Publisher’s Note

All claims expressed in this article are solely those of the authors and do not necessarily represent those of their affiliated organizations, or those of the publisher, the editors and the reviewers. Any product that may be evaluated in this article, or claim that may be made by its manufacturer, is not guaranteed or endorsed by the publisher.
