# Supplementary material for: Applicability of Small-Molecule Inhibitors in the Study of Peptidyl Arginine Deiminase 2 (PAD2) and PAD4
Source: Front Immunol. 2021 Oct 19;12:716250. doi: 10.3389/fimmu.2021.716250 (PMC8560728; doi:10.3389/fimmu.2021.716250)
Supplement: Supplementary file 1 [file DataSheet_1.docx]

Supplementary Material

##
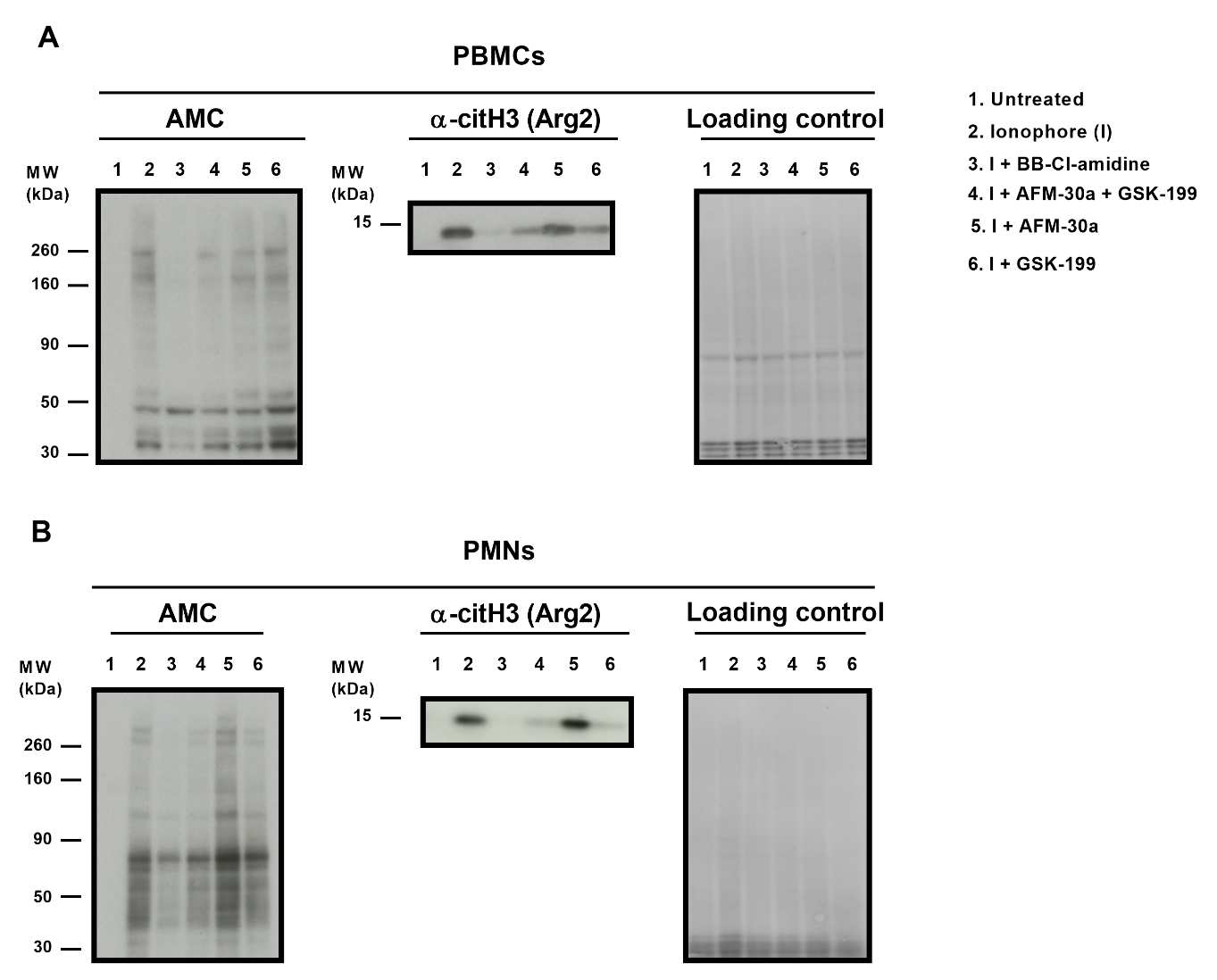
Supplementary Figures

**Supplementary figure 1. Inhibition of intracellular citrullination by small-molecule PAD inhibitors and loading controls.** Isolated PBMCs or PMNs from six healthy blood donors were pre-incubated with 20 μM of BB-Cl-amidine, 20 μM AFM-30a, 20 μM GSK199 or with a combination of the two latter for 1 h before addition of the calcium ionophore A23187. Untreated cells and ionophore-only treated cells (I) were used as negative and positive control, respectively. The cells were lysed, and the lysates were examined by Western blotting using the AMC method for visualisation of total citrullination, a mAb for detection of histone H3 citrullination or the Pierce™ Reversible Protein Stain Kit for PDVF Membranes as a control of total protein loading. **(A)** Representative AMC blot for PBMC lysates (left), blot for citrullinated histone H3 in PBMC lysates (middle) and total protein staining of the membrane where the PBMC lysates were transferred (right). **(B)** Representative AMC blot for PMN lysates (left), blot for citrullinated histone H3 in PMN lysates (middle) and total protein staining of the membrane where the PMNs lysates were transferred (right).


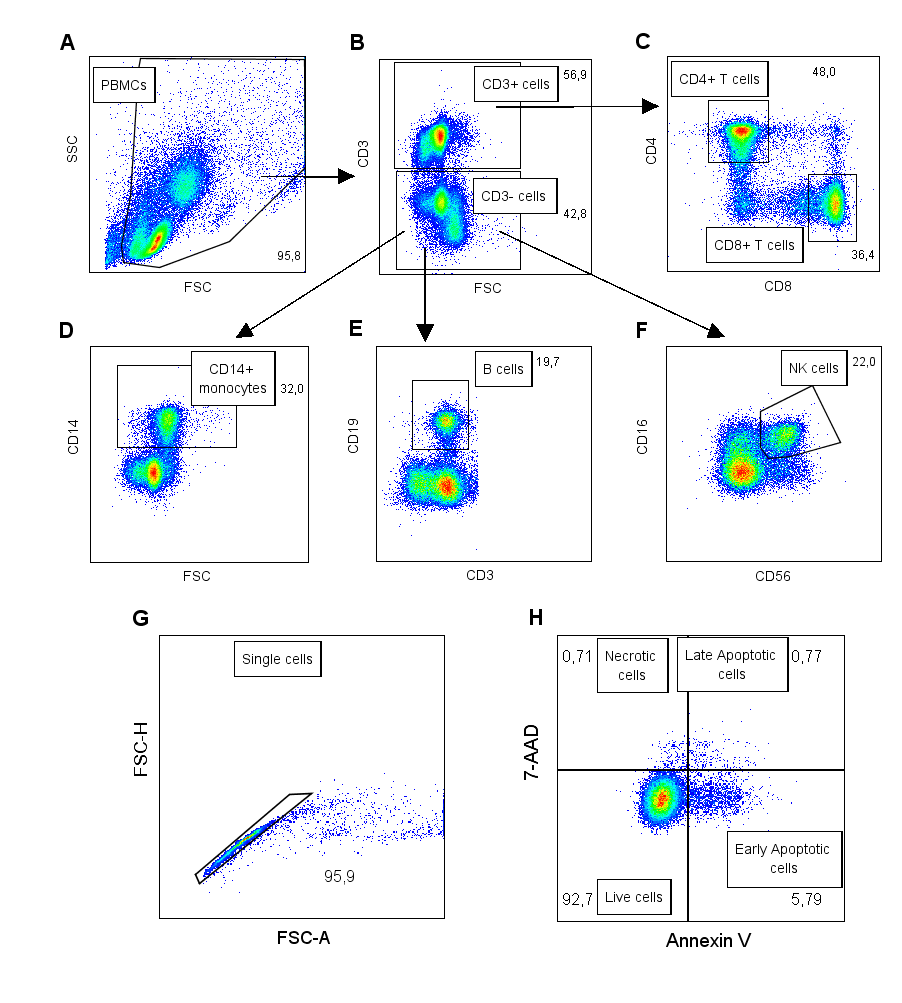


**Supplementary figure 2. Gating strategy for PBMC populations.** PBMCs from three healthy donors were cultured in presence of BB-Cl-amidine or a combination of AFM-30a and GSK199 at 1, 5, 10 and 20 μM concentrations. DMSO was used as vehicle control. After 24 h, 48 h and 72 h of incubation, the percentage of live and dead cells was assessed by flow cytometry after staining with Annexin V and 7-AAD. **(A)** Shows the whole PBMC gating, **(B)** shows the separation of PBMCs into CD3^+^ and CD3^-^ cells respectively, **(C)** shows the separation of CD3^+^ cells into CD3^+^ CD4^+^ T cells and CD3^+^ CD8^+^ T cells. Within the CD3^-^ PBMC gating, **(D)** shows the CD14^+^ monocytes, **(E)** shows the CD19^+^ B cells and **(F)** shows the CD16^+^ CD56^+^ NK cells. From all previously described populations doublets were excluded **(G)** and live and dead cells were determined by AnnexinV and 7-AAD staining as shown in **(H)**.


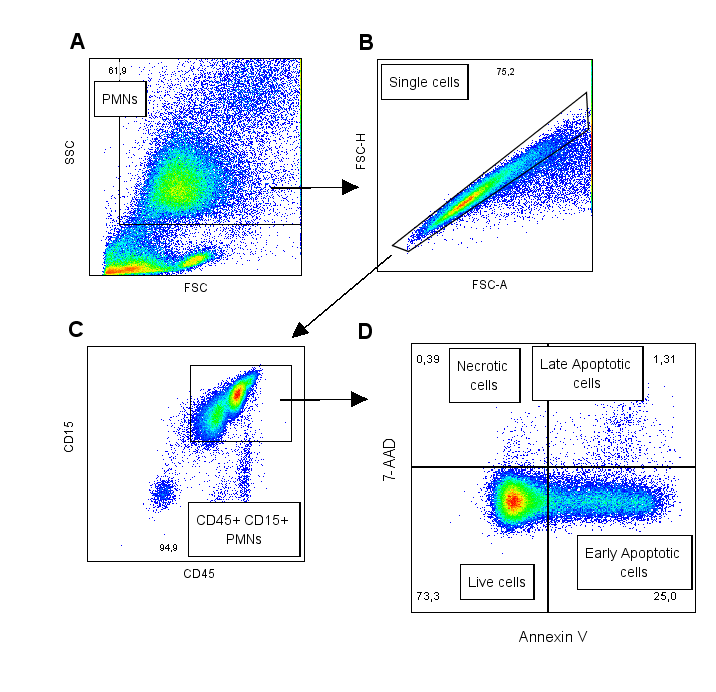


**Supplementary figure 3. Gating strategy for PMNs.** PMNs from three healthy donors were cultured in presence of BB-Cl-amidine or a combination of AFM-30a and GSK199 at 1, 5, 10 and 20 μM concentrations. DMSO was used as vehicle control. After 4 h and 24 h of incubation, the percentage of live and dead cells was assessed by flow cytometry after staining with Annexin V and 7-AAD. **(A)** Shows the whole PMN gating, **(B)** shows the exclusion of doublets and **(C)** shows the gating of CD45^+^ CD15^+^ PMNs. Live and dead cells were determined by AnnexinV and 7-AAD staining as shown in **(D)**.


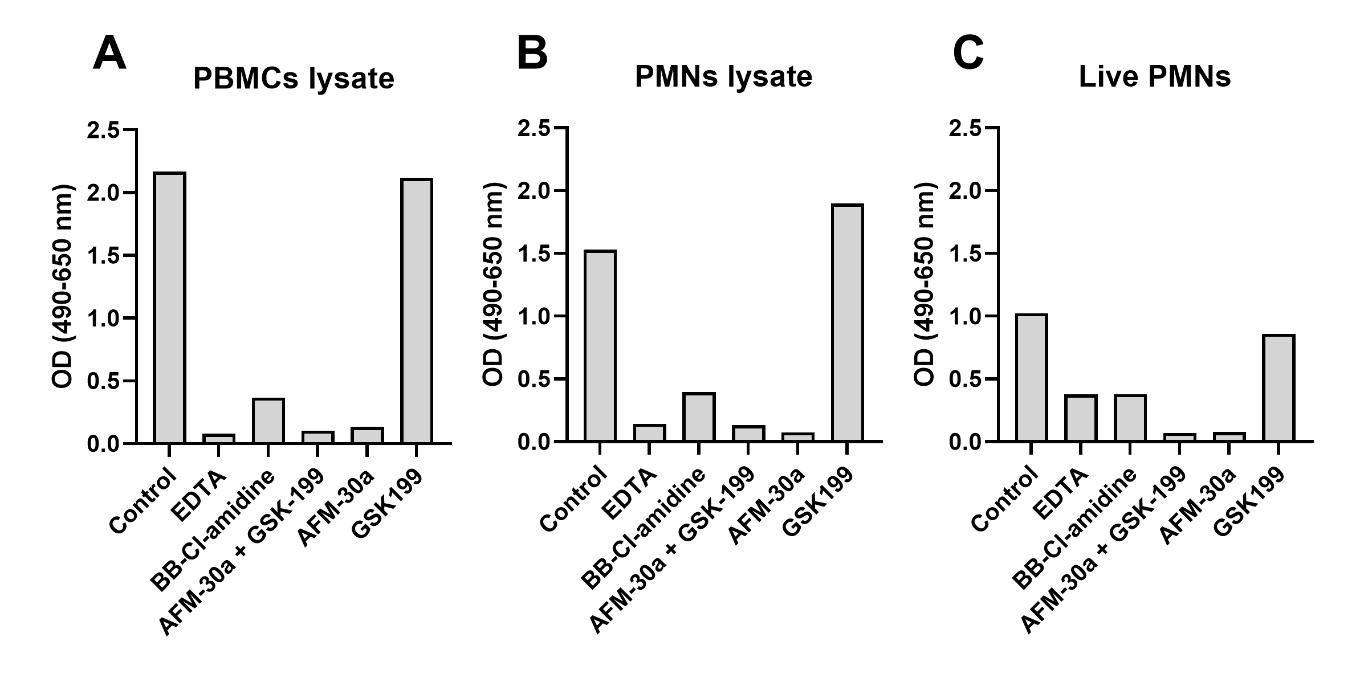


**Supplementary figure 4. Effect of PAD inhibitors on leukocyte-derived PADs and on PADs in RA synovial fluid.** ELISA plates were coated with human fibrinogen and incubated with **(A)** lysates from PBMCs, **(B)** lysates from PMNs, and **(C)** live PMNs. Incubation took place in the presence of 25 mM EDTA, 20 µM BB-Cl-amidine, 20 µM AFM-30a, 20 µM GSK199 or a combination of the latter two. Untreated lysates or live cells were used as controls. After 3 h of incubation, citrullination was detected with mAb 20B2 which recognizes a citrullinated epitope of fibrinogen. Raw OD values at 490-650 nm are shown for a representative donor.


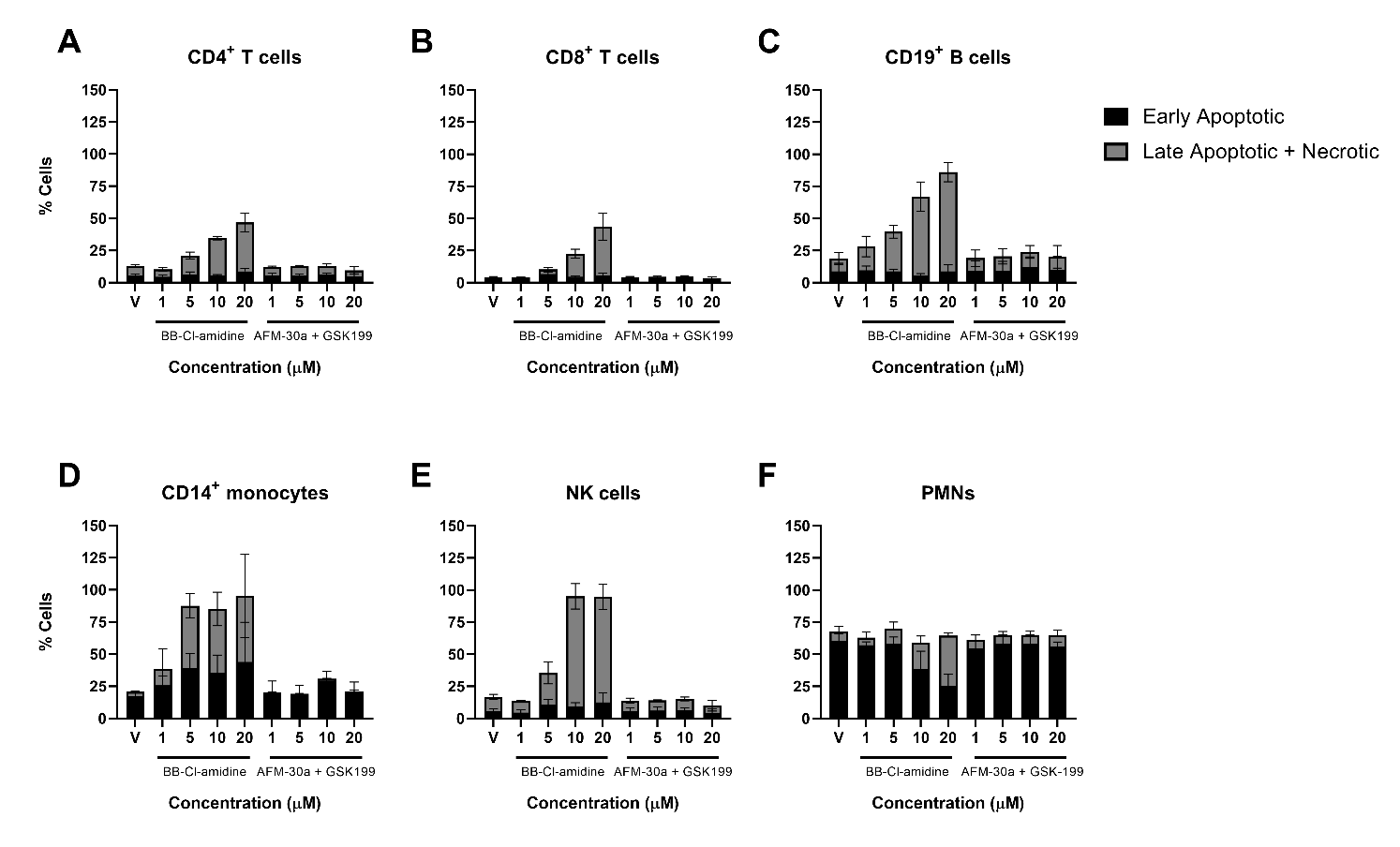


**Supplementary figure 5. Effect of BB-Cl-amidine, AFM-30a and GSK199 on cell survival.** PBMCs and PMNs from three healthy donors were cultured in presence of BB-Cl-amidine or a combination of AFM-30a and GSK199 at the given concentrations. DMSO was used as vehicle control (V). After 48 h of incubation, the percentage of live and dead cells was assessed by flow cytometry after staining with Annexin V and 7-AAD. The percentages of early apoptotic and dead cells are shown for **(A)** CD4^+^ T cells, **(B)** CD8^+^ T cells, **(C)** B cells, **(D)** CD14^+^ monocytes, and **(E)** NK cells. **(F)** A similar staining was performed for PMNs incubated for 24 h with the inhibitors. Bars and error bars indicate mean + SD.


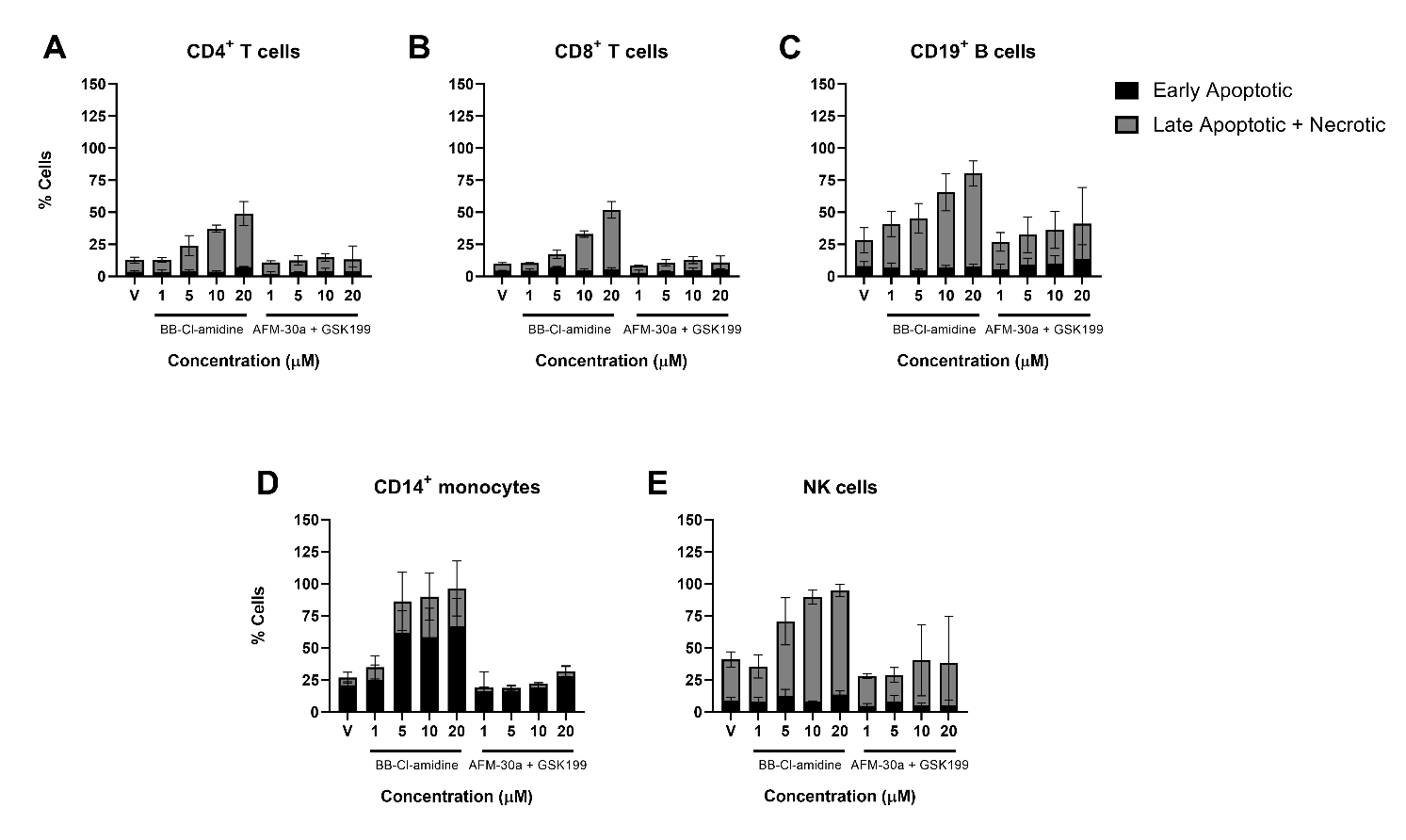


**Supplementary figure 6. Effect of BB-Cl-amidine, AFM-30a and GSK199 on cell survival.** PBMCs from three healthy donors were cultured in presence of BB-Cl-amidine or a combination of AFM-30a and GSK199 at the given concentrations. DMSO was used as vehicle control (V). After 72 h of incubation, the percentage of live and dead cells was assessed by flow cytometry after staining with Annexin V and 7-AAD. The percentages of early apoptotic and dead cells are shown for **(A)** CD4^+^ T cells, **(B)** CD8^+^ T cells, **(C)** B cells, **(D)** CD14^+^ monocytes, and **(E)** NK cells. Bars and error bars indicate mean + SD.
